# Assessment of Functional EST-SSR Markers (Sugarcane) in Cross-Species Transferability, Genetic Diversity among Poaceae Plants, and Bulk Segregation Analysis

**DOI:** 10.1155/2016/7052323

**Published:** 2016-06-01

**Authors:** Shamshad Ul Haq, Pradeep Kumar, R. K. Singh, Kumar Sambhav Verma, Ritika Bhatt, Meenakshi Sharma, Sumita Kachhwaha, S. L. Kothari

**Affiliations:** ^1^Biotechnology Division, UP Council of Sugarcane Research, Shahjahanpur 242001, India; ^2^Interdisciplinary Programme of Life Science for Advance Research and Education, University of Rajasthan, Jaipur 302004, India; ^3^Department of Botany, University of Rajasthan, Jaipur 302015, India; ^4^School of Biotechnology, Yeungnam University, Gyeongsan 712-749, Republic of Korea; ^5^Amity Institute of Biotechnology, Amity University Rajasthan, Jaipur 302006, India

## Abstract

Expressed sequence tags (ESTs) are important resource for gene discovery, gene expression and its regulation, molecular marker development, and comparative genomics. We procured 10000 ESTs and analyzed 267 EST-SSRs markers through computational approach. The average density was one SSR/10.45 kb or 6.4% frequency, wherein trinucleotide repeats (66.74%) were the most abundant followed by di- (26.10%), tetra- (4.67%), penta- (1.5%), and hexanucleotide (1.2%) repeats. Functional annotations were done and after-effect newly developed 63 EST-SSRs were used for cross transferability, genetic diversity, and bulk segregation analysis (BSA). Out of 63 EST-SSRs, 42 markers were identified owing to their expansion genetics across 20 different plants which amplified 519 alleles at 180 loci with an average of 2.88 alleles/locus and the polymorphic information content (PIC) ranged from 0.51 to 0.93 with an average of 0.83. The cross transferability ranged from 25% for wheat to 97.22% for* Schlerostachya*, with an average of 55.86%, and genetic relationships were established based on diversification among them. Moreover, 10 EST-SSRs were recognized as important markers between bulks of pooled DNA of sugarcane cultivars through BSA. This study highlights the employability of the markers in transferability, genetic diversity in grass species, and distinguished sugarcane bulks.

## 1. Introduction

Sugarcane is a bioenergy crop belonging to the genus* Saccharum* L. of the tribe Andropogoneae (family: Poaceae). This tribe comprises grass species which have high economic value. The noble sugarcane varieties are developed from interspecific hybridization of* Saccharum officinarum* L. (2*n* = 80) which has high sugar content with less disease tolerance and* Saccharum spontaneum* (2*n* = 40 to 120) which provides stress, disease tolerance, and high fiber content for biomass. The taxonomy and genetic constitution of sugarcane are complicated due to complex interspecific aneupolyploid genome which makes chromosome numbers range from 100 to 130 [1]. Moreover, six* Saccharum* spp. (*S. spontaneum, S. officinarum*,* S. robustum*,* S. edule*,* S. barberi,* and* S. sinense*) and four* Saccharum* related genera (*Erianthus, Miscanthus*,* Sclerostachya,* and* Narenga*) have purportedly undergone interbreeding, forming the “*Saccharum* complex” [2, 3]. The interbreeding has made their genome more complex and added to multigenic and/or multiallelic nature for most agronomic traits that made sugarcane breeding a more difficult task [4].

A vast array of genomic tools has been developed which has opened new ways to define the genetic architecture of sugarcane and helped to explore its functional system [1, 5]. Among the molecular markers, microsatellites are most favored for a variety of genetic applications due to their multiallelic nature, high reproducibility, cross transferability, codominant inheritance, abundance, and extensive genome coverage [6–8]. Microsatellites or simple sequences repeats (SSRs) are monotonous repetitions of very short (one to six) nucleotide motifs, which occur as interspersed repetitive elements in all eukaryotic and prokaryotic genomes. However, transcribed regions of the genome also contain enormous range of microsatellites that correspond to genic microsatellites or EST-SSRs. Therefore, expressed sequence tags (ESTs) are the short transcribed portions and involved in the variety of metabolic functions. The presence of the microsatellites in genes as well as ESTs unveils the biological significance of SSR distribution, expansion, and contraction on the function of the genes themselves [9].

Presently, huge amounts of expressed sequence tags have been deposited in public database (NCBI). In silico approaches to retrieve EST sequences from NCBI and functional annotations provide more constructive EST-SSRs or gene-based SSR (genic SSRs) marker development besides own EST libraries development. This method of the EST-SSR markers development provides the easiest way to reduce cost, time, and labours along with more meaningful marker identifications [10]. The presence of microsatellites in the genic region is found to be more conserved due to which they possess high reproducibility and high interspecific/intraspecific transferability. Hence, EST-SSR could be used for polymorphism, genetic diversity, cross transferability, and comparative mapping in different plant species. Accordingly, several genetic studies were done on sugarcane using microsatellite markers to decipher polymorphism, cross transferability, genetic diversity, informative marker detection through bulk segregation analysis (BSA), and comparative genomics [8, 11–13]. The objective of the present study was to retrieve EST sequences for more informative EST-SSR development and their genetic assessment within and across the taxa through cross transferability, genetic relationships, and bulk segregation analysis.

## 2. Materials and Methods

### 2.1. EST Sequences Retrieving, ESTs Assembling, and Microsatellites Identification

Total 10000 EST sequences of the* Saccharum* spp. were downloaded in Fasta format from National Centre for Biotechnology Information (NCBI) for microsatellites deciphering. Further, ESTs assembling was carried out using CAP3 programme (http://mobyle.pasteur.fr/cgi-bin/portal.py#forms::cap3) for minimization of sequences redundancy. Microsatellite identification was carried out using MISA software (http://pgrc.ipk-gatersleben.de/misa/) and the criteria for SSR detection were 6, 4, 3, 3, and 3 repeat units for di-, tri-, tetra-, penta-, and hexanucleotides, respectively. SSR primer pairs (forward and reverse) were designed for the selected EST sequences having microsatellites using online web tool, batch primer 3 pipeline [14].

### 2.2. EST-SSR Sequences Annotation

Assessment of EST sequences having SSR was done through blastn/blastx analysis for homology search and against nonredundant (nr) protein at the NCBI. Furthermore, functional annotation pipeline was also run at online tool for gene ontology (GO) which was intended for different GO functional classes like biological process, cellular component, and molecular function [15].

### 2.3. PCR Amplification and Electrophoresis

PCR reactions were carried out in a total of 10 *μ*L volume containing 25 ng template DNA, 1.0 *μ*L (10 pmol/*μ*L) of each forward and reverse primer, 100 mM of dNTPs, 0.5 U of* Taq* DNA polymerase, and 1.0 *μ*L of 10x PCR buffer with 2.5 mM of MgCl_2_. Amplification was performed in a thermal cycler (Bio-Rad) in the following conditions: initial denaturation at 94°C for 5 min followed by 30 amplification cycles of denaturation for 1 min at 94°C followed by annealing temperature (*T*_*a*_) for 1 min and then extension for 2 min at 72°C; final extension at 72°C for 7 min was allowed. The PCR conditions particularly the annealing temperatures (varying from 52°C to 58°C) for each primer were standardized and amplified products were stored at 4°C. The PCR products were analyzed on a 7% native PAGE in vertical gel electrophoresis unit (Bangalore Genei*™*) using TBE buffer. The sizes of amplified fragments were estimated using 50 bp DNA ladder (Fermentas). Gels were documented using ethidium bromide (EtBr) stained dye.

### 2.4. Evaluation of Saccharum EST-SSR across the Taxa through Cross Transferability

The cross transferability of* Saccharum* derived EST-SSR markers was evaluated among the 20 accessions comprising seven cereals (wheat, maize, barley, rice, pearl millet, oat, and* Sorghum*), four* Saccharum* related genera (*Erianthus*,* Miscanthus*,* Narenga*, and* Sclerostachya*), three* Saccharum* species (51NG56 (*S. robustum*), N58 (*S. spontaneum*), and two clones of* S. officinarum* (Bandjermasin Hitam and Gunjera)), and five* Saccharum* commercial cultivars (CoS 88230, CoS 92423, UP 9530, CoS 8436, and CoS 91230). All genotypes were collected from the Sugarcane Research Institute Farm, UPCSR, Shahjahanpur, India. Furthermore, genomic DNA from young juvenile, disease-free, immature leaves was isolated for each genotype using CTAB (cetyl trimethylammonium bromide) method [16]. Isolated DNA samples were treated with RNAase for 1 h at 37°C and purified by phenol extraction (25 phenol : 24 chloroform : 1 isoamyl alcohol, v/v/v) followed by ethanol precipitation [17] and stored at −80°C. DNA was quantified on 0.8% agarose gel and the working concentration of 25 ng/*μ*L was obtained by making final adjustment in 10 mM TE buffer.

### 2.5. Genetic Diversity Analysis

The assessment of EST-SSRs in genetic diversity analysis was done among 20 plants belonging to distinct groups comprising cereals,* Saccharum* related genera,* Saccharum* species, and* Saccharum* cultivars. The allelic data of 63 EST-SSR primers were used to ascertain the genetic relationships between 20 genotypes by clustering analysis. Amplified bands were scored as binary data in the form of present (1) or absent (0). Dendrogram was constructed by neighbour-joining and Jaccard's algorithm using FreeTree and TreeView software [18, 19]. The polymorphic information content (PIC) values were calculated for each primer by using the online resource of PIC Calculator (http://www.liv.ac.uk/~kempsj/pic.html).

### 2.6. Informative Assessment of Functional EST-SSR Markers between Bulks

Plant materials were used as F2 mapping population comprising 209 genotypes of the sugarcane cultivars which were developed from cross between CoS 91230 (Parent; CoS 775 × Co 1148) with CoS 8436 (Parent; MS 68/47 × Co 1148) from September to March (2010-2011). Grouping of genotypes was done according to their stem diameter (contrasting high and low stem diameter genotypes) into two sets. DNA extractions were carried out from both sets and equal quantities of genomic DNA from 10 extreme high stem diameter and 10 extreme low stem diameter genotypes were pooled into two bulks. PCR amplification was done in both bulks with newly developed EST-SSR primers for informative markers identifications through bulk segregation analysis (BSA) [20].

## 3. Results and Discussion

### 3.1. Mining of Microsatellites in EST Sequences and SSRs Characterization

Total 10,000 EST sequences related to* Saccharum* spp. were examined from NCBI for the simple sequence repeat (SSR) identification and characterization using computational approach. Prior to the marker deciphering, sequence assembly was performed and 6201 (4201 kb) nonredundant sequences were detected comprising 1752 contigs and 4449 singlets, wherein 406 SSRs were identified with 360 perfect SSRs and 37 sequences containing more than 1 SSR and 30 SSRs in compound formation. Therefore, computational and experimental approach to ascertain microsatellites in EST libraries from public database (NCBI) turned to be very cost effective and reduces time and labour besides expense of own libraries development. EST-SSRs are a more preferable DNA marker in the variety of genetic analysis and found to be more conserved as present in the transcribed region of the genome. These were found to be more transferable across the taxonomic boundaries and could be evaluated as most informative markers for variety of genomics applications [10, 21]. These are more adapted in plants comparative genetic analysis for gene identification, gene mapping, marker-assisted-selection, transferability, and genetic diversity [7, 22–24]. Also, a variety of studies have been reported on sugarcane using EST-SSR markers for desired genetic analysis [8, 13, 25, 26].

The frequency of SSR in EST sequences was 6.4% including all the repeats except mononucleotide repeats. This result is comparatively higher compared to previous studies on sugarcane [8, 27–29]. Contrary to this, Singh et al. [13] reported higher frequency (9.3%) in sugarcane. Kumpatla and Mukhopadhyay [30] also observed high range (2.65% to 10.62%) of SSR frequency in different plant species. In general, about 5% of ESTs contained SSR which has been reported in many plant species [31]. These variations in microsatellite frequency could be attributed to the “search criteria” used, type of SSR motif, size of sequence data, and the mining tools used [24, 32]. In other words, the density of the microsatellites was one SSR per 10.45 kb which is closely comparable to earlier studies in sugarcane with densities 1 SSR/10.9 kb [8] and 1/9 kb SSR [13].

**Figure 1:**
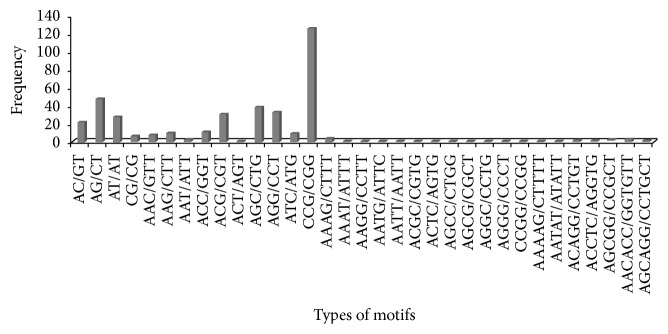
Details of 33 different types of nucleotide repeat motifs belonging to di-, tri-, tetra-, penta-, and hexanucleotide repeat motifs with sequence complementarity.

**Figure 2:**
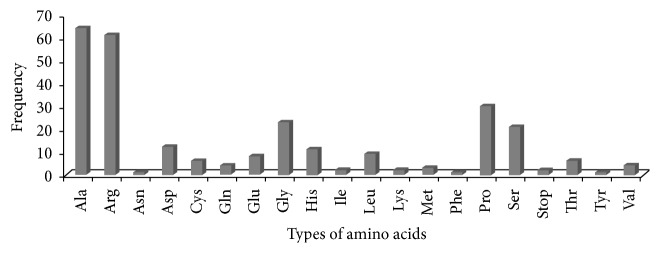
Details of different types of predicted amino acids encoded by trinucleotide repeat motifs.

Analysis revealed that trinucleotide repeats (66.74%) were found to be more frequent followed by di- (26.10%), tetra- (4.67%), penta- (1.5%), and hexanucleotide (1.2%) repeats. Our observation of high frequency of trinucleotide repeats is in agreement with previous reports on sugarcane [8, 13, 27–29, 33]. Several other studies have also represented high frequency of trinucleotide repeats in different plant species [24, 31, 34–36]. A total of 33 different types of motifs were identified of which four belonged to dinucleotide, eight belonged to trinucleotides, twelve belonged to tetranucleotide, five belonged to pentanucleotide, and two belonged to hexanucleotide repeats (Figure 1). We observed that motifs AG/CT and AT/AT were more frequent in dinucleotide repeat followed by motifs CCG/CGG, AGC/CTG, AGG/CCT, and ACG/CGT in trinucleotide repeat, motif AAAG/CTTT in tetranucleotide repeats, motif ACAGG/CCTGT in pentanucleotide repeats, and AACACC/GGTGTT in hexanucleotide repeats. The presence of motif CCG/CGG was also observed in sugarcane by different authors [13, 27]. Kantety et al. [37] also reported CCG/CGG motif as most abundant in wheat and* Sorghum*. Similarly, both Lawson and Zhang [38] and Da Maia et al. [39] also observed abundance of motif CCG/CGG in different member of the grass family. Victoria et al. [35] also decoded motif CCG/CGG in the lower plants (*C. reinhardtii* and* P. patens*). Thus, this predominance of CCG/CGG motif frequency has been related to a high GC-content [5]. Some motifs which are responsible for making unusual DNA folding structure (hairpin formed, bipartite triplex formed, and simple loop folding) also have effect on gene expressions and regulations mechanism, namely, CCT/AGG, CCG/GGC, GGA/TTC, and GAA/TTC motifs [40, 41]. Moreover, the presence of trinucleotide repeats in the coding region formed a distinct group and encoded amino acid tracts within the peptide [42]. We also observed predictable twenty different types of amino acids including stop codon. Alanine, arginine, glycine, proline, and serine were most frequent (Figure 2). This is in agreement with previous studies that reported on different plant species [11, 35, 43].

### 3.2. Expressed Sequence Tags Annotation and Primers Development

**Figure 3:**
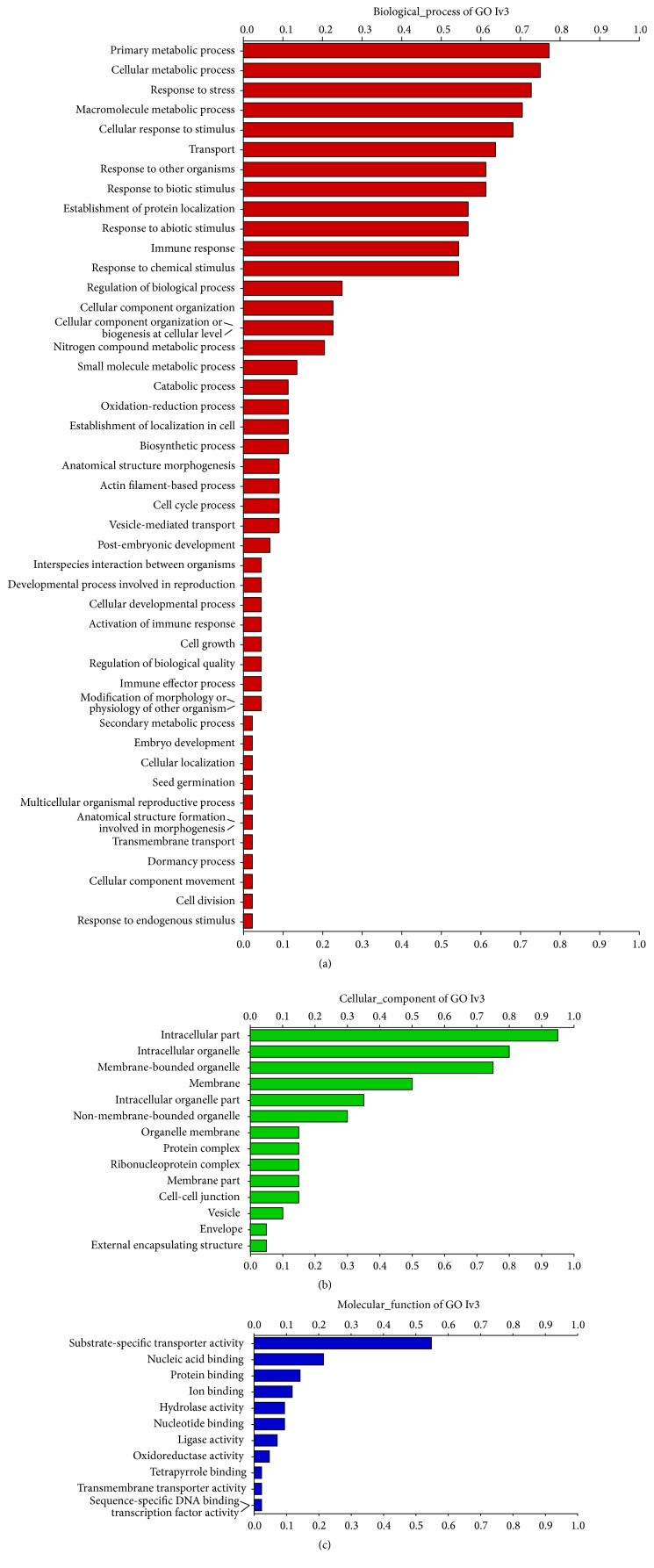
Most promising results of gene ontology (GO) as horizontal bar graphs. These graphs represent the distribution of GO terms categorized as a biological process (a), cellular component (b), and molecular function (c).

All EST sequences having SSRs were examined by functional annotation (blastn, blastx, and gene ontology). After-effect, sixty-three ESTs having SSRs were successfully identified on the basis of their involvement in the various metabolic processes (Figure 3). After-effect, sixty-three EST-SSRs primer pairs were designed for polymorphic nature, cross transferability, bulk segregation analysis, and genetic diversity in the test plants (Table 1). These selected EST-SSRs comprised all types of repeat motifs (excluding mononucleotide repeat), and among trinucleotide repeats they were highly frequent with GCT/CGA, TCC/AGG, and GGT/CCA repeat motifs. Similarly, Sharma et al. [44] also used functional annotation pipelines for the more prominent molecular markers development related to gene transcripts. Selected EST-SSRs were associated with various pathways of metabolic process, namely, GO:0006281 DNA repair, GO:0006301 postreplication repair, GO:0016070 RNA metabolic process, GO:0016070 RNA metabolic process, GO:0006446 regulation of translational initiation, GO:0015991 ATP hydrolysis coupled proton transport, GO:0006629 lipid metabolic process, GO:0015031 protein transport, GO:0005667 transcription factor complex, GO:0005815 microtubule organizing centre, GO:0003743 translation initiation factor activity, GO:0017005 3′-tyrosyl-DNA phosphodiesterase activity, GO:0030042 actin filament depolymerization, and GO:0015078 hydrogen ion transmembrane transporter activity, and so forth (see the complete details of the most promising hits of gene ontology of EST-SSRs in the supplementary table available online at http://dx.doi.org/10.1155/2016/7052323).

### 3.3. Assessment of EST-SSR Marker in Selected Plants

**Figure 4:**
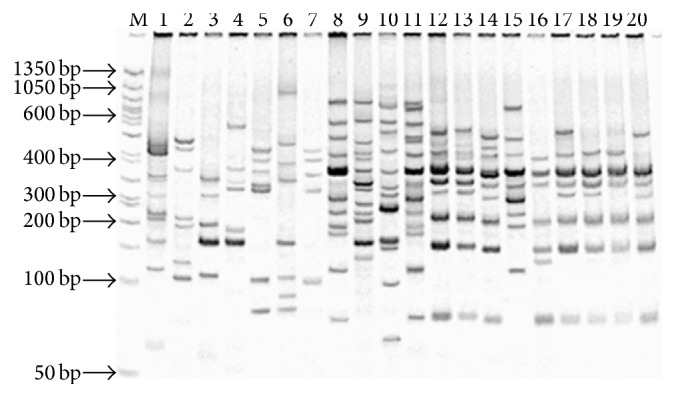
The gel represents PCR amplification profile with SYMS37 primer among twenty different plant species. Lanes: 1* wheat*, 2* maize*, 3* barley*, 4* rice*, 5* pearl millet*, 6* oat*, 7* Sorghum*, 8* Narenga*, 9* Schlerostachya*, 10* Erianthus*, 11* Miscanthus*, 12* Bandjermasin Hitam*, 13* Gunjera*, 14* 51NG56*, 15* N58*, 16* CoS 92423*, 17* CoS 88230*, 18* UP 9530*, 19* CoS 91230*, and 20* CoS 8436*.

A set of 63 EST-SSR primers were evaluated for PCR optimization, polymorphism, and cross amplification in twenty genotypes belonging to cereals plants and* Saccharum* related genera and* Saccharum* species and their commercial cultivars, of which 42 EST-SSR primers produced successful amplifications with both expected and unexpected sizes (Figure 4). Among 42 EST-SSRs, twenty-eight belonged to trinucleotide repeats with then seven of tetra-, three of penta-, three of hexa-, and one of dinucleotide repeats. Meanwhile, PCR amplifications produced 519 alleles (expected size) at 180 loci with an average of 2.88 alleles per locus. This result is comparable with earlier studies that reported on various plant species, namely, 2.79 alleles/locus in rice varieties [45], 2.9 to 6.0 alleles per locus in maize [46], and 3.04 alleles/locus in rye [47]. However, our result of alleles per locus is lower compared to previous studies that reported on sugarcane, that is, 6.04 alleles/locus [28], 7.55 alleles/locus [29], and 6.0 alleles/locus [48]. The polymorphic information content (PIC) was extended from 0.51 to 0.93 with an average of 0.83. It could be encompassed that low and high range of allelic amplifications with EST-SSRs correspond to marker polymorphism and low level of polymorphism from EST-SSRs might be due to possible selection against alterations in the conserved sequences of EST-SSRs [49, 50].

### 3.4. Cross Transferability

The potentials of EST-SSR primers were examined for cross transferability among 20 plant species belonging to cereals and* Saccharum* related genera and* Saccharum* species and their cultivars under the same PCR conditions. However, 42 EST-SSRs showed successful amplifications among all the selected plants. The cross transferability was estimated to be 27.22% in wheat, 27.22% in maize, 47.22% in barely, 46.66% in rice, 36.11% in pearl millet, 55.55% in oat, 26.11% in* Sorghum*, 88.33% in* Narenga*, 98.88% in* Sclerostachya*, 71.11% in* Erianthus*, 60.0% in* Miscanthus*, 73.33% in* Bandjermasin Hitam*, 55.55% in* Gunjera*, 75.55% in 51NG56, 55.0% in N58, 50.56% in CoS 92423, 58.88% in CoS 88230, 51.11% in UP 9530, 52.78% in CoS 91230, and 60.0% in CoS 8436. Meanwhile, the frequency distributions of cross transferability of EST-SSRs ranged from 26.11% for* Sorghum* to 98.88% for* Sclerostachya*, with an average of 55.86% (Table 2).* Saccharum* related genera (79.58%) and* Saccharum* species (64.86%) showed high rate of cross transferability compared to other groups. This is in agreement with previous studies reported on* Saccharum* species and* Saccharum* related genera [12, 13, 51]. Several earlier studies related to cross transferability have been reported on distinct plant groups from different families using EST-SSRs markers [7, 52, 53]. This suggests that transferring ability of genic markers makes it compatible to determine genetic studies across the taxa for utilization in mapping of genes from related species along with genera and identification of suspended hybridization. This can also aid vigilance of the introgression of genetic entity from wild relatives to cultivated, comparative mapping and establishing evolutionary relationship between them. Thus, microsatellites derived from expressed region of the genome are expected to be more conserved and more transferable across taxa.

### 3.5. Genetic Diversity Analysis by EST-SSRs

**Figure 5:**
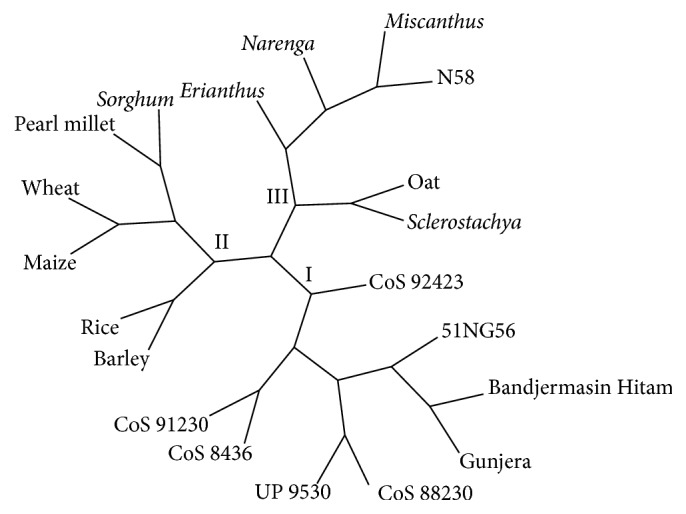
Dendrogram is constructed based on allelic data produced from 42 EST-SSR markers using FreeTree and TreeView software.

In order to evaluate the potential of EST-SSRs, the genetic analysis was done among 20 genotypes belonging to 7 cereals (wheat, maize, barley, rice, pearl millet, oat, and* Sorghum*), 4* Saccharum* related genera (*Erianthus*,* Miscanthus*,* Narenga*, and* Sclerostachya*), 3* Saccharum* species (51NG56 (*S. robustum*), N58 (*S. spontaneum*), and two of* S. officinarum* clones (*Gunjera* and* Bandjermasin Hitam*)), and 5 sugarcane commercial cultivars (CoS 8436, CoS 91230, CoS 88230, UP 9530, and CoS 92423). The generated allelic data were used for genetic relationships analysis by making dendrogram based on Jaccard's and neighbour-joining algorithm using FreeTree and TreeView software. The dendrogram fell into three major clusters with several edges, cluster I with eight genotypes comprising most of* Saccharum* species and their commercial cultivars, cluster II encompassing six genotypes of most of cereals species, and cluster III with six species comprising most of the* Saccharum* related genera along with some interventions (Figure 5). This relationship is in agreement with previous studies reported by other authors [12, 13, 51, 54]. Our EST-SSRs markers showed close syntenic relationship and their evolutionary nature among the 20 genotypes into three major clusters with some genotypes divergence. These relationships have resulted from the expansion and contraction of SSRs in conserved EST sequences within the same group of plant species along with some variation having resulted from higher evolutionary divergence among them. Several earlier studies also reported on genetic diversity analysis within and across the plant taxa using molecular marker [7, 8, 24, 48, 52, 55–57]. Thus, microsatellite markers distinguished all the genotypes to certain extent and also provided the realistic estimate of genetic diversity among them.

### 3.6. Bulk Segregation Analysis (BSA) in Sugarcane

**Figure 6:**
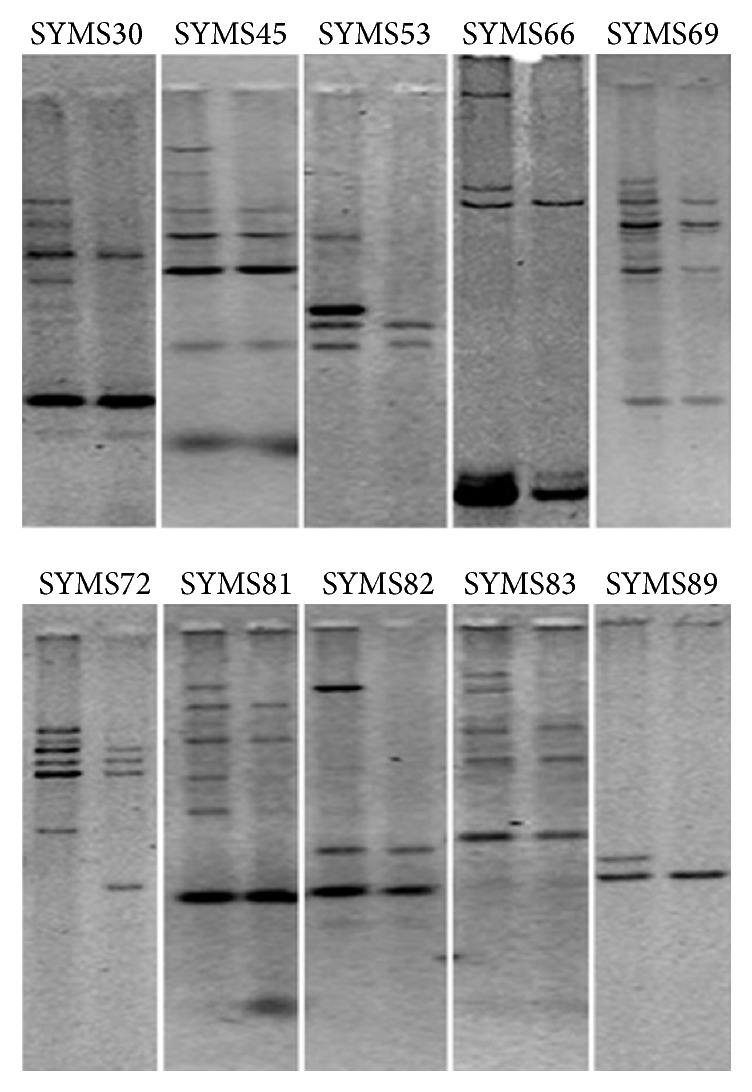
The gel represents polymorphism and discrimination between bulks of pooled DNA with contrasting high and low plant diameter through bulk segregation analysis.

All the 42 EST-SSR markers were evaluated in pooled DNA bulks of contrasting trait of sugarcane cultivars (CoS 91230 (CoS 775 × Co 1148) cross with CoS 8436 (MS 68/47 × Co 1148)) for the identification of reporter EST-SSR markers based on their allelic differences between them. Interestingly, 10 markers showed polymorphic nature and apparently discriminating potential between bulks through bulk segregation analysis (Figure 6). Among these, markers SYMS30, SYMS53, SYMS82, and SYMS89 showed a better response to discriminating the bulks. BSA is the strategy that involves the identification of genetic markers associated with character or trait which are based on their allelic differences between bulks [20]. Earlier studies have been established in sugarcane for the most prominent molecular markers detection linked to desirable traits through BSA. For example, molecular markers apparently linked to high fiber content in* Saccharum* species [58–60] and molecular markers used for QTL analysis and utilized for generating genetic maps around resistance genes in sugarcane against diseases and pests through BSA [12, 61, 62]. Several other studies also reported on selection of different agronomic traits in sugarcane for breeding programme with the development of molecular markers through BSA [1, 63–65]. Alternatively, BSA approach has been recently used for various purposes against the identification of differential expressed gene associated with both qualitative and quantitative using of the cDNA-AFLP approach [66–69]. Thus, BSA approach provides the easiest way in the direction of trait linked marker identification and also makes it possible to select informative markers beside evaluations of each marker in the whole progeny.

## 4. Conclusion

The present study was intended for identification and characterization of SSR in* Saccharum* spp. expressed sequence tag which is retrieved from public database (NCBI). Further, functional annotation was feasible to identify the most eminent EST-SSR markers selection. Therefore, this is the bypass way for EST-SSR markers development which reduces cost and time and provides an efficient way to analyze the transcribed portion of genome besides expense of own libraries development. A total of 63 EST-SSR markers were developed and experimentally validated for cross transferability along with their genetic relationships and also used for differentiation between pooled DNA bulks of* Saccharum* cultivars. These markers showed successful transferability rate among the twenty genotypes and established genetic diversity among cereals,* Saccharum* species/cultivars, and* Saccharum* related genera with some inconsistency. Further, some prominent marker also distinguished pooled DNA bulks of sugarcane cultivars based on stem diameter. Consequently, these EST-SSR markers were found to be more convenient which made it easy for us to use them as informative markers in further genetic studies in sugarcane breeding programme.

## Supplementary Material

Supplementary Table: Brief details of best promising hits obtained through gene ontology for ESTs having SSR which were categorized into biological process, cellular component and molecular function.

## Figures and Tables

**Table 1:** Details of selected 63 EST-SSR primer pairs used for cross transferability, genetic diversity, and bulks segregation analysis.

Serial number	Type	Primer sequence	Annealing temperature	SSR motif	PIC value	*E*-value	Putative identities (blastn/blastx)
SYMS28	F	GCGTCAGAGTGTTAAAACAAG	53	(GCT)_4_	0.81	9.39*E* − 42	Protein transport protein Sec61 beta
SYMS28	R	GTGTAGAACTGGAGCATTGAG					
SYMS29	F	GGGCAAGCAAGAAACCAC	52	(TCC)_4_	0.91	1.62*E* − 24	Protein translation factor SUI1
SYMS29	R	GAAGAGGTCAACCAAGAACTC					
SYMS30	F	GCGTCAGAGTGTTAAAACAAG	53	(GCT)_4_	0.86	1.00*E* − 21	Preprotein translocase Sec
SYMS30	R	GTGTAGAACTGGAGCATTGAG					
SYMS31	F	GAAGCTCCCAAGCTGCTA	53	(AGCT)_3_	0.76	2.00*E* − 12	*Predicted*: uncharacterized protein
SYMS31	R	CCTACAGGAAAGATTTTAGGG					
SYMS32	F	GTCTCTTCTCCAGTTCTCCTT	55	(TGCG)_4_	0.84	2.46*E* − 63	*Predicted*: actin-depolymerizing factor
SYMS32	R	GCTCAACAAATGTCTCCCTA					
SYMS33	F	TGCACTAACATGGTTGATGT	54	(GAAG)_3_	0.86	2.82*E* − 90	Hypothetical protein SORBIDRAFT_03g046450
SYMS33	R	GGTGATTGTAAGGGTCATCTT					
SYMS34	F	GTTAATGGTGGTTCCGTTC	53	(GGC)_6_	0.88	4*E* − 20	*Predicted*: uncharacterized protein LOC101783547
SYMS34	R	ATTATCAGCGCAGAGACATC					
SYMS35	F	GCGTCAGAGTGTTAAAACAAG	52	(GCT)_4_	0.75	1.00*E* − 21	Preprotein translocase
SYMS35	R	GTGTAGAACTGGAGCATTGAG					
SYMS36	F	GGACTGTACAAGGACGACAG	53	(GCT)_4_	0.70	1.14*E* − 41	Protein transport protein Sec61 beta subunit
SYMS36	R	TCTGCTTTCTTGGATATGGTA					
SYMS37	F	AAGAAGGATGCAAAGAAGAAG	54	(GAT)_4_	0.90	3.08*E* − 81	Hypothetical protein SORBIDRAFT_03g046450
SYMS37	R	AGGCTTAGTAACAGCAGGTTT					
SYMS38	F	AAGAAGGATGCAAAGAAGAAG	56	(AGA)_4_	0.86	9.00*E* − 37	Hypothetical protein
SYMS38	R	AGGCTTAGTAACAGCAGGTTT					
SYMS39	F	GGACTGTACAAGGACGACAG	—	(GCT)_4_	—	1.14*E* − 41	Protein transport protein
SYMS39	R	TCTGCTTTCTTGGATATGGTA					
SYMS40	F	GGACTGTACAAGGACGACAG	—	(GCT)_4_	—	1.25*E* − 40	Preprotein translocase
SYMS40	R	TCTGCTTTCTTGGATATGGTA					
SYMS41	F	GGACTGTACAAGGACGACAG	—	(GCT)_4_	—	9.62*E* − 42	Protein transport protein Sec61 beta subunit
SYMS41	R	TCTGCTTTCTTGGATATGGTA					
SYMS42	F	CCAAAGAGATCTTGCAGACTA	—	(ATG)_4_	—	1.78*E* − 53	Jasmonate-induced protein
SYMS42	R	CCCAACACAACAACCAAT					
SYMS43	F	CCACACAAGCAAGAAATAAAC	—	(GGT)_4_	—	8.57*E* − 74	Dirigent-like protein
SYMS43	R	TCGAACACTATGGTAAAGGTG					
SYMS44	F	GGACTGTACAAGGACGACAG	—	(GCT)_4_	—	1.15*E* − 41	Homeodomain-like transcription factor
SYMS44	R	TCTGCTTTCTTGGATATGGTA					
SYMS45	F	GCGTCAGAGTGTTAAAACAAG	53	(GCT)_4_	0.69	9.86*E* − 42	Protein transport protein
SYMS45	R	GACTCTGCTTTCTTGGATATG					
SYMS46	F	AGCTATCTTTAGTGGGGACAT	52	(CGT)_4_	0.90	1.82*E* − 44	Hypothetical protein SORBIDRAFT_09g006220
SYMS46	R	GAGGTCTCATCGGAGCTTA					
SYMS47	F	AGGTCGTTTTAATTCCTTCC	53	(GTTTT)_3_	0.77	1.00*E* − 21	Preprotein translocase Sec
SYMS47	R	CGTAAATATGAACGAGGTCAG					
SYMS48	F	AGGTCGTTTTAATTCCTTCC	53	(TTTA)_6_	0.90	4.00*E* − 20	TPA: hypothetical protein
SYMS48	R	CGTAAATATGAACGAGGTCAG					
SYMS49	F	GGACTGTACAAGGACGACAG	—	(GCT)_4_	—	1.15*E* − 41	Zinc finger A20 and AN1 domains-containing protein
SYMS49	R	TCTGCTTTCTTGGATATGGTA					
SYMS50	F	TCCAAGGATTTAGCTATGGAT	—	(TGT)_10_	—	6.79*E* − 13	TPA: seed maturation protein
SYMS50	R	TTCAACTACACCCTTCTGTTG					
SYMS51	F	GCGTCAGAGTGTTAAAACAAG	—	(GCT)_4_	—	1.22*E* − 41	Hypothetical protein
SYMS51	R	ATTGTCACTTGCTATCCATTT					
SYMS52	F	CACCTTCTTTCCTTCTCCTC	—	(CGC)_4_	—	3.32*E* − 47	V-type proton ATPase 16 kDa proteolipid subunit
SYMS52	R	GTAGATACCGAGCACACCAG					
SYMS53	F	TCAGTTCAGGGATGACAATAG	56	(CCGTGG)_3_	0.87	2.59*E* − 78	Homeodomain-like transcription factor superfamily protein
SYMS53	R	GGATAGACTGAAATCTGCTCA					
SYMS54	F	CAACTCGACTCTTTTCTCTCA	—	(CTC)_5_	—	4.13*E* − 08	Protein transport protein SEC31
SYMS54	R	GGAGGTGGAACTTCCTGA					
SYMS55	F	GGACTGTACAAGGACGACAG	—	(GCT)_4_	—	1.12*E* − 41	Protein transport protein Sec61 subunit beta-like isoform
SYMS55	R	TCTGCTTTCTTGGATATGGTA					
SYMS56	F	GGACTGTACAAGGACGACAG	—	(GCT)_4_	—	8.01*E* − 42	Protein transport protein Sec61 subunit beta-like isoform
SYMS56	R	TCTGCTTTCTTGGATATGGTA					
SYMS57	F	AAACGATCAGATACCGTTGTA	—	(CG)_6_	—	7.84*E* − 27	Caltractin
SYMS57	R	ATCAAAGAGATCAAAGGCTTC					
SYMS58	F	CATTTCGAAGCTCCTCCT	52	(CCTCCG)_6_	0.74	5.97*E* − 66	Zinc finger A20 and AN1 domains-containing protein
SYMS58	R	TAGGCTGCACAACAATAGTCT					
SYMS59	F	CTCCCCCATTTCTCTTCC	53	(GCAGCC)_6_	0.80	4.02*E* − 65	*Predicted*: reticulon-like protein B1
SYMS59	R	CAAGTACTCCAGCAGAGATGT					
SYMS60	F	CTTTTCCCTCTTCCTCTCTC	—	(CCG)_5_	—	1.24*E* − 45	*Predicted*: uncharacterized tRNA-binding protein
SYMS60	R	TGTCACTAACACGAATCACAA					
SYMS61	F	CCCTCTCCCTGCTCTTTC	54	(TCC)_5_	0.79	4.14*E* − 57	Actin-depolymerizing factor 3
SYMS61	R	CAGTCACAAAGTCGAAATCAT					
SYMS62	F	ACAACTCTTCAGTCTTCACGA	54	(CAAC)_3_	0.85	4.40*E* − 66	Truncated alcohol dehydrogenase
SYMS62	R	CCAATCTTGACATCCTTGAC					
SYMS63	F	GCACGGTGAAGTTCTAGTTC	54	(TCGAT)_4_	0.67	3.11*E* − 31	Hypothetical protein SORBIDRAFT_08g002800
SYMS63	R	CAGCTTCACTCATGAATTTTT					
SYMS64	F	GGACTGTACAAGGACGACAG	—	(GCT)_4_	—	1.08*E* − 41	Protein transport protein Sec61 subunit beta-like isoform
SYMS64	R	TCTGCTTTCTTGGATATGGTA					
SYMS65	F	AACACAAGCAAGAAATAAACG	53	(GGT)_4_	0.51	3.42*E* − 74	Dirigent-like protein
SYMS65	R	AACACTATGGTCAAGGTGGTA					
SYMS66	F	GCGTCAGAGTGTTAAAACAAG	52	(GCT)_4_	0.58	1.01*E* − 41	Protein transport protein Sec61 subunit beta-like isoform
SYMS66	R	GAAATCGCTCTATAAGGTTCC					
SYMS67	F	TCTCTCTGAAGATGATGCTTT	52	(AAG)_5_	0.90	4.25*E* − 83	Hypothetical protein SORBIDRAFT_03g005100
SYMS67	R	GTTAAGAGGCTTCCAAAGAAC					
SYMS68	F	CAGCTCGTCGTCTTCTTTT	—	(GTC)_5_		2.00*E* − 55	Putative ubiquitin-conjugating enzyme family
SYMS68	R	GTGGCTTGTTTGGATATTCTT					
SYMS69	F	GGACTGTACAAGGACGACAG	54	(GCT)_4_	0.79	9.28*E* − 42	Protein transport protein Sec61 subunit beta-like isoform
SYMS69	R	CGTCAGACGTACTGAAATGTT					
SYMS70	F	AACACAAGCAAGAAATAAACG	53	(GGT)_4_	0.77	1.58*E* − 73	Putative dirigent protein
SYMS70	R	AACACTATGGTCAAGGTGGTA					
SYMS71	F	GGACTGTACAAGGACGACAG	—	(GCT)_4_	—	9.86*E* − 42	Protein transport protein Sec61 subunit beta-like isoform
SYMS71	R	TCTGCTTTCTTGGATATGGTA					
SYMS72	F	CCCTCTCCCTGCTCTTTC	55	(TCC)_4_	0.89	4.36*E* − 57	Actin-depolymerizing factor 3
SYMS72	R	CAGTCACAAAGTCGAAATCAT					
SYMS73	F	GGACTGTACAAGGACGACAG	55	(GCT)_4_	0.83	1.09*E* − 41	Protein transport protein Sec61 subunit beta-like isoform
SYMS73	R	TCTGCTTTCTTGGATATGGTA					
SYMS74	F	GGACTGTACAAGGACGACAG	52	(GCT)_4_	0.88	9.51*E* − 42	Preprotein translocase Sec
SYMS74	R	TCTGCTTTCTTGGATATGGTA					
SYMS75	F	GCACCCCCAATTCGAACG	52	(ACG)_3_	0.93	1.78*E* − 68	TPA: general regulatory factor 1
SYMS75	R	CGGTAGTCCTTGATGAGTGT					
SYMS76	F	GGACTGTACAAGGACGACAG	52	(GCT)_4_	0.78	4.79*E* − 41	Protein transport protein Sec61 subunit beta-like isoform
SYMS76	R	TCTGCTTTCTTGGATATGGTA					
SYMS77	F	CACGCAACGCAAGCACAG	55	(CCAT)_3_	0.93	8.34*E* − 70	Hypothetical protein SORBIDRAFT_10g030160
SYMS77	R	AAGTTGATTCACCCTCATTCT					
SYMS78	F	CACGCAACGCAAGCACAG	53	(CGATC)_3_	0.92	1.04*E* − 41	Translocon-associated protein alpha subunit precursor
SYMS78	R	AAGTTGATTCACCCTCATTCT					
SYMS79	F	GGACTGTACAAGGACGACAG	53	(GCT)_4_	0.91	1.04*E* − 41	Protein transport protein Sec61 subunit beta-like isoform
SYMS79	R	TCTGCTTTCTTGGATATGGTA					
SYMS80	F	CTTGATCCTTGACAAAAGAGA	52	(AG)_6_	0.87	2.25*E* − 59	*Predicted*: ubiquitin-conjugating enzyme E2
SYMS80	R	ATTGCTGTTGATATTTGGATG					
SYMS81	F	GCGTCAGAGTGTTAAAACAAG	53	(GCT)_4_	0.87	8.01*E* − 42	Protein transport protein Sec61 subunit beta-like isoform
SYMS81	R	GTGTAGAACTGGAGCATTGAG					
SYMS82	F	TATCAACAAGCCTTCCATTC	53	(GTG)_4_	0.90	1.12*E* − 30	Glycine-rich RNA-binding protein 2
SYMS82	R	GGCTATAGTCACCACGGTAG					
SYMS83	F	CGACAGGGAGAAGAGTACAG	55	(GCT)_4_	0.87	9.39*E* − 42	Protein transport protein Sec61 subunit beta-like isoform
SYMS83	R	GACTCTGCTTTCTTGGATATG					
SYMS84	F	GCGTCAGAGTGTTAAAACAAG	53	(GCT)_4_	0.75	1.14*E* − 41	Protein transport protein Sec61 subunit beta-like isoform
SYMS84	R	AATCGCTCTATAAGGTTCCTC					
SYMS85	F	CTCTTCTTCACCAATTCCTCT	—	(CCG)_6_	—	1.14*E* − 51	Protein transport protein Sec61 subunit beta-like isoform
SYMS85	R	CAAACCTCATAAAGAGTGCAG					
SYMS86	F	GGGCAAGCAAGAAACCAC	54	(TCC)_4_	0.93	1.16*E* − 28	TPA: translation initiation factor 1
SYMS86	R	CGTACATGAACGTAGTCCTTT					
SYMS87	F	GCGTCAGAGTGTTAAAACAAG	—	(GCT)_4_	—	1.19*E* − 41	Protein transport protein Sec61 beta subunit
SYMS87	R	AATCGCTCTATAAGGTTCCTC					
SYMS88	F	TTATAAGGAAATCCCCCACT	—	(GCC)_4_	—	7.71*E* − 55	Hypothetical protein SORBIDRAFT_09g000970
SYMS88	R	CACCAAGTACTCATCCATCAT					
SYMS89	F	CATCTCCTGCTAACAATTCAC	55	(TGC)_4_	0.91	9.64*E* − 60	*Predicted*: NAC domain-containing protein
SYMS89	R	ATTTATAGGTTGGCACCAGAG					
SYMS90	F	GCGTCAGAGTGTTAAAACAAG	53	(GCT)_4_	0.85	1.00*E* − 22	Protein transport protein Sec61 subunit beta-like isoform
SYMS90	R	GTGTAGAACTGGAGCATTGAG					

**Table 2:** Details of cross transferability of 42 EST-SSR markers in twenty genotypes belonging to cereals and *Saccharum* related genera and *Saccharum* species and their cultivars. Lanes 1 to 20 represent number of bands produced in wheat, maize, barley, rice, pearl millet, oat, *Sorghum*, *Narenga*, *Sclerostachya*, *Erianthus*, *Miscanthus*, *Bandjermasin Hitam*, *Gunjera*, 51NG56, N58, CoS 92423, CoS 88230, UP 9530, CoS 91230, and CoS 8436, respectively.

S. number/lane	1	2	3	4	5	6	7	8	9	10	11	12	13	14	15	16	17	18	19	20	Primer polymorphism (%)
SY 28	4	5	6	1	2	3	0	2	5	5	3	2	3	2	3	3	1	3	2	3	95
SY 29	6	3	5	9	3	12	3	5	12	6	7	7	8	7	7	6	7	6	6	8	100
SY 30	1	0	4	0	0	7	0	5	6	6	3	3	4	3	2	3	2	3	2	3	80
SY 31	0	0	0	0	0	0	0	2	3	2	1	1	2	2	2	0	2	2	0	0	50
SY 32	1	0	0	1	0	2	0	2	4	3	1	3	0	0	1	0	0	0	0	0	55
SY 33	0	0	2	2	3	5	3	7	6	4	6	5	5	5	2	3	0	2	3	3	85
SY 34	0	0	0	0	0	0	0	0	0	0	0	3	2	5	4	5	5	5	9	9	85
SY 35	0	0	0	1	0	4	0	5	2	1	0	0	1	1	1	2	2	1	2	1	65
SY 36	0	0	0	0	0	0	0	0	3	0	0	5	2	2	2	0	2	0	0	2	35
SY 37	9	7	5	6	6	5	5	9	12	10	11	8	8	8	8	6	7	6	5	6	100
SY 38	0	0	0	3	0	2	0	7	8	1	0	0	2	2	1	2	0	0	3	0	50
SY 45	0	0	0	0	0	2	0	5	1	0	1	2	1	1	0	0	0	0	0	0	35
SY 46	0	1	4	4	1	0	0	4	4	0	1	0	0	0	0	0	0	0	0	0	35
SY 47	0	1	3	3	2	0	0	1	0	0	0	0	0	0	0	0	0	0	0	0	25
SY 48	0	0	0	0	5	3	0	8	12	0	5	3	3	2	1	3	2	2	0	0	60
SY 53	0	0	0	1	1	3	3	12	9	2	1	1	2	2	1	3	2	1	3	1	85
SY 58	0	0	0	1	1	0	3	2	1	0	0	0	0	0	0	0	0	0	0	0	25
SY 59	0	0	0	0	0	0	0	4	0	3	3	0	0	1	0	0	0	0	0	0	20
SY 61	0	3	7	5	4	0	3	4	5	6	3	4	1	5	3	5	5	5	5	5	90
SY 62	0	0	0	1	0	2	0	4	5	3	2	2	0	0	0	0	0	0	0	0	35
SY 63	0	0	0	0	0	0	0	1	1	4	1	6	1	1	1	0	1	0	2	1	55
SY 65	0	0	0	0	0	0	0	0	0	1	1	1	0	1	2	3	2	2	2	1	50
SY 66	0	0	0	0	0	0	0	1	0	0	0	3	1	2	0	0	3	2	2	1	40
SY 67	1	2	5	6	3	3	0	2	5	6	3	2	0	0	2	0	0	0	0	0	60
SY 69	1	5	2	0	1	0	0	3	1	2	1	1	1	1	1	2	1	0	3	2	80
SY 70	1	0	0	1	0	1	0	5	2	0	0	1	0	0	2	0	3	3	0	0	45
SY 72	1	0	2	0	0	0	0	1	4	5	0	8	5	8	1	3	6	4	6	8	70
SY 73	0	0	2	0	1	2	0	2	2	0	1	3	1	4	1	0	3	0	0	3	60
SY 74	2	1	1	0	1	1	3	1	3	2	1	4	1	5	1	0	3	0	0	2	80
SY 75	1	0	2	3	3	0	0	0	6	3	9	7	6	7	6	0	4	6	0	0	65
SY 76	0	0	0	0	0	2	0	1	2	1	4	0	0	0	1	0	0	0	0	0	30
SY 77	2	5	6	6	8	4	3	9	6	9	3	0	4	4	3	4	3	0	2	0	85
SY 78	2	2	5	6	4	6	4	5	7	6	4	10	9	10	3	2	8	7	5	9	100
SY 79	0	0	0	0	1	0	0	3	5	2	0	5	1	7	7	2	1	0	0	6	55
SY 80	1	1	0	4	0	1	0	4	5	6	2	5	4	5	5	3	5	4	5	5	85
SY 81	0	0	0	1	2	6	0	6	6	8	7	3	3	4	3	3	3	3	3	2	80
SY 82	0	0	10	3	1	4	3	9	1	1	6	5	4	3	5	3	4	4	3	2	90
SY 83	1	2	0	6	2	3	4	2	7	2	1	3	2	3	1	3	3	2	3	3	95
																					
SY 84	0	0	0	1	2	3	0	0	2	0	0	3	3	1	0	3	3	3	3	3	60
SY 86	1	5	6	3	2	5	4	5	8	11	6	5	3	11	5	12	7	6	7	10	100
SY 89	9	6	5	4	1	7	3	7	4	3	10	4	4	5	7	3	4	7	3	5	100
SY 90	5	0	3	2	5	2	3	4	3	4	0	4	3	6	4	4	2	3	6	4	90
Average of transferability	27.22	27.22	47.22	46.67	36.11	55.56	26.11	88.33	98.89	71.11	60.0	73.33	55.56	75.56	55.0	50.56	58.89	51.11	52.78	60.0	
