# Supplementary material for: Assessment of Functional EST-SSR Markers (Sugarcane) in Cross-Species Transferability, Genetic Diversity among Poaceae Plants, and Bulk Segregation Analysis
Source: Genet Res Int. 2016 Jun 1;2016:7052323. doi: 10.1155/2016/7052323 (PMC4908241; doi:10.1155/2016/7052323)
Supplement: Supplementary file 1 — Supplementary Table: Brief details of best promising hits obtained through gene ontology for ESTs having SSR which were categorized into biological process, cellular component and molecular function. [file 7052323.f1.pdf]

Supplementary Table: Brief details of best promising hits obtained through gene ontology for ESTs having SSR which were categorized into biological process, cellular component and molecular function.

Serial no.	Biological Process	Cellular Component	Molecular Function
SYMS-29	GO:0006281 DNA repair<br>GO:0009616 virus induced gene silencing<br>GO:0010050 vegetative phase change<br>GO:0006446 regulation of translational initiation	GO:0005634 nucleus<br>GO:0005840 ribosome	GO:0003743 translation initiation factor activity<br>GO:0017005 3'-tyrosyl-DNA phosphodiesterase activity
SYMS-32	GO:0030042 actin filament depolymerization	GO:0005737 cytoplasm<br>GO:0015629 actin cytoskeleton	GO:0003779 actin binding
SYMS-33		GO:0005886 plasma membrane	
SYMS-36	GO:0009627 systemic acquired resistance<br>GO:0006457 protein folding<br>GO:0009644 response to high light intensity<br>GO:0034976 response to endoplasmic reticulum stress<br>GO:0042542 response to hydrogen peroxide<br>GO:0009408 response to heat<br>GO:0015031 protein transport		GO:0008565 protein transporter activity
SYMS-37,38	GO:0016070 RNA metabolic process<br>GO:0006446 regulation of translational initiation	GO:0005634 nucleus<br>GO:0005840 ribosome	GO:0005525 GTP binding<br>GO:0003743 translation initiation factor activity
SYMS-39	GO:0009627 systemic acquired resistance<br>GO:0006457 protein folding<br>GO:0009644 response to high light intensity<br>GO:0034976 response to endoplasmic reticulum stress<br>GO:0042542 response to hydrogen peroxide<br>GO:0009408 response to heat<br>GO:0015031 protein transport		GO:0008565 protein transporter activity
SYMS-40	GO:0009627 systemic acquired resistance<br>GO:0006457 protein folding<br>GO:0009644 response to high light intensity<br>GO:0034976 response to endoplasmic reticulum stress<br>GO:0042542 response to hydrogen peroxide<br>GO:0009408 response to heat<br>GO:0015031 protein transport		GO:0008565 protein transporter activity
SYMS-41	GO:0009627 systemic acquired resistance<br>GO:0006457 protein folding<br>GO:0009644 response to high light intensity<br>GO:0034976 response to endoplasmic		GO:0008565 protein transporter activity

	reticulum stress<br>GO:0042542 response to hydrogen peroxide<br>GO:0009408 response to heat<br>GO:0015031 protein transport		
SYMS-44	GO:0009627 systemic acquired resistance<br>GO:0006457 protein folding<br>GO:0009644 response to high light intensity<br>GO:0034976 response to endoplasmic reticulum stress<br>GO:0042542 response to hydrogen peroxide<br>GO:0009408 response to heat<br>GO:0015031 protein transport		GO:0008565 protein transporter activity
SYMS-45	GO:0009627 systemic acquired resistance<br>GO:0006457 protein folding<br>GO:0009644 response to high light intensity<br>GO:0034976 response to endoplasmic reticulum stress<br>GO:0042542 response to hydrogen peroxide<br>GO:0009408 response to heat<br>GO:0015031 protein transport		GO:0008565 protein transporter activity
SYMS-46			GO:0046983 protein dimerization activity
SYMS-49	GO:0009627 systemic acquired resistance<br>GO:0006457 protein folding<br>GO:0009644 response to high light intensity<br>GO:0034976 response to endoplasmic reticulum stress<br>GO:0042542 response to hydrogen peroxide<br>GO:0009408 response to heat<br>GO:0015031 protein transport		GO:0008565 protein transporter activity
SYMS-51	GO:0009627 systemic acquired resistance<br>GO:0006457 protein folding<br>GO:0009644 response to high light intensity<br>GO:0034976 response to endoplasmic reticulum stress<br>GO:0042542 response to hydrogen peroxide<br>GO:0009408 response to heat<br>GO:0015031 protein transport		GO:0008565 protein transporter activity
SYMS-52	GO:0015991 ATP hydrolysis coupled proton transport	GO:0016023 cytoplasmic membrane-bounded vesicle<br>GO:0005886 plasma membrane<br>GO:0033179 proton-transporting V-type ATPase, V0 domain<br>GO:0005774 vacuolar membrane<br>GO:0016021 integral to membrane	GO:0015078 hydrogen ion transmembrane transporter activity

SYMS-53	GO:0006355 regulation of transcription, DNA-dependent	GO:0005634 nucleus<br>GO:0005667 transcription factor complex	GO:0003700 sequence-specific DNA binding transcription factor activity<br>GO:0043565 sequence-specific DNA binding
SYMS-55	GO:0009627 systemic acquired resistance<br>GO:0006457 protein folding<br>GO:0009644 response to high light intensity<br>GO:0034976 response to endoplasmic reticulum stress<br>GO:0042542 response to hydrogen peroxide<br>GO:0009408 response to heat<br>GO:0015031 protein transport		GO:0008565 protein transporter activity
SYMS-56	GO:0009627 systemic acquired resistance<br>GO:0006457 protein folding<br>GO:0009644 response to high light intensity<br>GO:0034976 response to endoplasmic reticulum stress<br>GO:0042542 response to hydrogen peroxide<br>GO:0009408 response to heat<br>GO:0015031 protein transport		GO:0008565 protein transporter activity
SYMS-57	GO:0055114 oxidation-reduction process<br>GO:0006281 DNA repair<br>GO:0060151 peroxisome localization<br>GO:0051301 cell division<br>GO:0007067 mitosis<br>GO:0051646 mitochondrion localization<br>GO:0051645 Golgi localization<br>GO:0030048 actin filament-based movement	GO:0005815 microtubule organizing center<br>GO:0005737 cytoplasm<br>GO:0005886 plasma membrane	GO:0003676 nucleic acid binding<br>GO:0005509 calcium ion binding<br>GO:0008026 ATP-dependent helicase activity<br>GO:0005524 ATP binding<br>GO:0032440 2-alkenal reductase [NAD(P)] activity
SYMS-58	GO:0048573 photoperiodism, flowering<br>GO:0007067 mitosis<br>GO:0006950 response to stress		GO:0003677 DNA binding<br>GO:0008270 zinc ion binding
SYMS-59	GO:0071786 endoplasmic reticulum tubular network organization	GO:0005773 vacuole<br>GO:0071782 endoplasmic reticulum tubular network<br>GO:0009506 plasmodesma<br>GO:0005886 plasma membrane<br>GO:0071458 integral to cytosolic side of endoplasmic reticulum membrane	
SYMS-60	GO:0034968 histone lysine methylation<br>GO:0009909 regulation of flower development<br>GO:0009640 photomorphogenesis<br>GO:0009220 pyrimidine ribonucleotide biosynthetic process<br>GO:0010388 cullin deneddylation<br>GO:0000085 G2 phase of mitotic cell cycle	GO:0005829 cytosol<br>GO:0009506 plasmodesma	GO:0004812 aminoacyl-tRNA ligase activity<br>GO:0000049 tRNA binding

	GO:0006418 tRNA aminoacylation for protein translation		
SYMS-61	GO:0030042 actin filament depolymerization<br>GO:0016049 cell growth<br>GO:0006623 protein targeting to vacuole<br>GO:0042742 defense response to bacterium<br>GO:0000902 cell morphogenesis<br>GO:0009870 defense response signaling pathway, resistance gene-dependent<br>GO:0048193 Golgi vesicle transport	GO:0015629 actin cytoskeleton<br>GO:0009507 chloroplast<br>GO:0005634 nucleus<br>GO:0005886 plasma membrane	GO:0003779 actin binding
SYMS-62	GO:0006116 NADH oxidation<br>GO:0000947 amino acid catabolic process to alcohol via Ehrlich pathway<br>GO:0043458 ethanol biosynthetic process involved in glucose fermentation to ethanol<br>GO:0006090 pyruvate metabolic process	GO:0005739 mitochondrion<br>GO:0005634 nucleus	GO:0008270 zinc ion binding<br>GO:0019170 methylglyoxal reductase (NADH-dependent) activity<br>GO:0004022 alcohol dehydrogenase (NAD) activity
SYMS-63	GO:0006629 lipid metabolic process		
SYMS-64	GO:0009627 systemic acquired resistance<br>GO:0006457 protein folding<br>GO:0009644 response to high light intensity<br>GO:0034976 response to endoplasmic reticulum stress<br>GO:0042542 response to hydrogen peroxide<br>GO:0009408 response to heat<br>GO:0015031 protein transport		GO:0008565 protein transporter activity
SYMS-66	GO:0009627 systemic acquired resistance<br>GO:0006457 protein folding<br>GO:0009644 response to high light intensity<br>GO:0034976 response to endoplasmic reticulum stress<br>GO:0042542 response to hydrogen peroxide<br>GO:0009408 response to heat<br>GO:0015031 protein transport		GO:0008565 protein transporter activity
SYMS-67	GO:0001510 RNA methylation<br>GO:0009220 pyrimidine ribonucleotide biosynthetic process	GO:0005829 cytosol<br>GO:0009507 chloroplast<br>GO:0005773 vacuole<br>GO:0005634 nucleus	GO:0005515 protein binding
SYMS-68	GO:0006511 ubiquitin-dependent protein catabolic process<br>GO:0009910 negative regulation of flower development<br>GO:0033523 histone H2B ubiquitination<br>GO:0009650 UV protection<br>GO:0009965 leaf morphogenesis		GO:0004842 ubiquitin-protein ligase activity

	GO:0010228 vegetative to reproductive phase transition of meristem		
SYMS-69	GO:0009627 systemic acquired resistance<br>GO:0006457 protein folding<br>GO:0009644 response to high light intensity<br>GO:0034976 response to endoplasmic reticulum stress<br>GO:0042542 response to hydrogen peroxide<br>GO:0009408 response to heat<br>GO:0015031 protein transport		GO:0008565 protein transporter activity
SYMS-71	GO:0009627 systemic acquired resistance<br>GO:0006457 protein folding<br>GO:0009644 response to high light intensity<br>GO:0034976 response to endoplasmic reticulum stress<br>GO:0042542 response to hydrogen peroxide<br>GO:0009408 response to heat<br>GO:0015031 protein transport		GO:0008565 protein transporter activity
SYMS-72	GO:0030042 actin filament depolymerization<br>GO:0016049 cell growth<br>GO:0006623 protein targeting to vacuole<br>GO:0042742 defense response to bacterium<br>GO:0000902 cell morphogenesis<br>GO:0009870 defense response signaling pathway, resistance gene-dependent<br>GO:0048193 Golgi vesicle transport	GO:0015629 actin cytoskeleton<br>GO:0009507 chloroplast<br>GO:0005634 nucleus<br>GO:0005886 plasma membrane	GO:0003779 actin binding
SYMS-73	GO:0009627 systemic acquired resistance<br>GO:0006457 protein folding<br>GO:0009644 response to high light intensity<br>GO:0034976 response to endoplasmic reticulum stress<br>GO:0042542 response to hydrogen peroxide<br>GO:0009408 response to heat<br>GO:0015031 protein transport		GO:0008565 protein transporter activity
SYMS-74	GO:0009627 systemic acquired resistance<br>GO:0006457 protein folding<br>GO:0009644 response to high light intensity<br>GO:0034976 response to endoplasmic reticulum stress<br>GO:0042542 response to hydrogen peroxide<br>GO:0009408 response to heat<br>GO:0015031 protein transport		GO:0008565 protein transporter activity
SYMS-75	GO:0009793 embryo development ending in seed dormancy	GO:0005773 vacuole<br>GO:0005618 cell wall	GO:0016818 hydrolase activity, acting on acid anhydrides, in phosphorus-containing anhydrides

	GO:0009740 gibberellic acid mediated signaling pathway<br>GO:0016570 histone modification<br>GO:0006355 regulation of transcription, DNA-dependent<br>GO:0009407 toxin catabolic process<br>GO:0048449 floral organ formation<br>GO:0043090 amino acid import<br>GO:0009965 leaf morphogenesis<br>GO:0010265 SCF complex assembly<br>GO:0016567 protein ubiquitination<br>GO:0006486 protein glycosylation<br>GO:0009845 seed germination<br>:0006888 ER to Golgi vesicle-mediated transport<br>GO:0046686 response to cadmium ion<br>GO:0031146 SCF-dependent proteasomal ubiquitin-dependent protein catabolic process<br>GO:0009737 response to abscisic acid stimulus<br>GO:0009909 regulation of flower development<br>GO:0010162 seed dormancy	GO:0005794 Golgi apparatus<br>GO:0005829 cytosol<br>GO:0019005 SCF ubiquitin ligase complex<br>GO:0005635 nuclear envelope<br>GO:0009570 chloroplast stroma<br>GO:0009506 plasmodesma<br>GO:0005886 plasma membrane<br>GO:0005739 mitochondrion	GO:0019904 protein domain specific binding<br>GO:0005524 ATP binding<br>GO:0045309 protein phosphorylated amino acid binding
SYMS-76	GO:0009627 systemic acquired resistance<br>GO:0006457 protein folding<br>GO:0009644 response to high light intensity<br>GO:0034976 response to endoplasmic reticulum stress<br>GO:0042542 response to hydrogen peroxide<br>GO:0009408 response to heat<br>GO:0015031 protein transport		GO:0008565 protein transporter activity
SYMS-77,78		GO:0016023 cytoplasmic membrane-bounded vesicle<br>GO:0005783 endoplasmic reticulum	
SYMS-79	GO:0009627 systemic acquired resistance<br>GO:0006457 protein folding<br>GO:0009644 response to high light intensity<br>GO:0034976 response to endoplasmic reticulum stress<br>GO:0042542 response to hydrogen peroxide<br>GO:0009408 response to heat<br>GO:0015031 protein transport		GO:0008565 protein transporter activity
SYMS-80	GO:0051788 response to misfolded protein<br>GO:0006635 fatty acid beta-oxidation<br>GO:0043161 proteasomal ubiquitin-dependent protein catabolic process<br>GO:0016579 protein deubiquitination	GO:0005737 cytoplasm<br>GO:0005886 plasma membrane<br>GO:0005634 nucleus	GO:0004842 ubiquitin-protein ligase activity

	):0016558 protein import into peroxisome matrix<br>GO:0051510 regulation of unidimensional cell growth<br>GO:0048193 Golgi vesicle transport<br>GO:0042023 DNA endoreduplication<br>GO:0016567 protein ubiquitination<br>GO:0006301 postreplication repair<br>GO:0043248 proteasome assembly		
SYMS-81	GO:0015031 protein transport<br>GO:0009627 systemic acquired resistance<br>GO:0034976 response to endoplasmic reticulum stress		GO:0008565 protein transporter activity
SYMS-82	GO:0009631 cold acclimation	GO:0005829 cytosol<br>GO:0005634 nucleus	GO:0003723 RNA binding<br>GO:0000166 nucleotide binding<br>GO:0008270 zinc ion binding
SYMS-83	GO:0009627 systemic acquired resistance<br>GO:0006457 protein folding<br>GO:0009644 response to high light intensity<br>GO:0034976 response to endoplasmic reticulum stress<br>GO:0042542 response to hydrogen peroxide<br>GO:0009408 response to heat<br>GO:0015031 protein transport		GO:0008565 protein transporter activity
SYMS-84	GO:0009627 systemic acquired resistance<br>GO:0006457 protein folding<br>GO:0009644 response to high light intensity<br>GO:0034976 response to endoplasmic reticulum stress<br>GO:0042542 response to hydrogen peroxide<br>GO:0009408 response to heat<br>GO:0015031 protein transport		GO:0008565 protein transporter activity
SYMS-85	GO:0006301 postreplication repair<br>GO:0006850 mitochondrial pyruvate transport<br>GO:0009060 aerobic respiration<br>GO:0006096 glycolysis<br>GO:0046686 response to cadmium ion	GO:0005743 mitochondrial inner membrane<br>GO:0005774 vacuolar membrane<br>GO:0009507 chloroplast<br>GO:0005886 plasma membrane	
SYMS-86	GO:0006281 DNA repair<br>GO:0009616 virus induced gene silencing<br>GO:0010050 vegetative phase change<br>GO:0006446 regulation of translational initiation	GO:0005634 nucleus<br>GO:0005840 ribosome	GO:0003743 translation initiation factor activity<br>GO:0017005 3'-tyrosyl-DNA phosphodiesterase activity
SYMS-87	GO:0009627 systemic acquired resistance<br>GO:0006457 protein folding<br>GO:0009644 response to high light intensity<br>GO:0034976 response to endoplasmic		GO:0008565 protein transporter activity

	reticulum stress<br>GO:0042542 response to hydrogen peroxide<br>GO:0009408 response to heat<br>GO:0015031 protein transport		
SYMS-88	GO:0043447 alkane biosynthetic process<br>GO:0022900 electron transport chain<br>GO:0042742 defense response to bacterium<br>GO:0019745 pentacyclic triterpenoid biosynthetic process<br>GO:0016126 sterol biosynthetic process	GO:0016021 integral to membrane<br>GO:0005789 endoplasmic reticulum membrane	GO:0046872 metal ion binding<br>GO:0020037 heme binding
SYMS-89	GO:0006355 regulation of transcription, DNA-dependent		GO:0003677 DNA binding
